# CO-releasing Metal Carbonyl Compounds as Antimicrobial Agents in the Post-antibiotic Era*[Fn FN2]

**DOI:** 10.1074/jbc.R115.642926

**Published:** 2015-06-08

**Authors:** Lauren K. Wareham, Robert K. Poole, Mariana Tinajero-Trejo

**Affiliations:** From the Department of Molecular Biology and Biotechnology, The University of Sheffield, Sheffield S10 2TN, United Kingdom

**Keywords:** antibiotic resistance, bacterial metabolism, carbon monoxide, heme, heme oxygenase, metal homeostasis, respiratory chain, transport metal, antimicrobial agents, metal carbonyl compound

## Abstract

The possibility of a “post-antibiotic era” in the 21st century, in which common infections may kill, has prompted research into radically new antimicrobials. CO-releasing molecules (CORMs), mostly metal carbonyl compounds, originally developed for therapeutic CO delivery in animals, are potent antimicrobial agents. Certain CORMs inhibit growth and respiration, reduce viability, and release CO to intracellular hemes, as predicted, but their actions are more complex, as revealed by transcriptomic datasets and modeling. Progress is hindered by difficulties in detecting CO release intracellularly, limited understanding of the biological chemistry of CO reactions with non-heme targets, and the cytotoxicity of some CORMs to mammalian cells.

## Introduction

It is axiomatic that metal ions are essential in biology, but also toxic in unregulated concentrations or locations. A corollary is that selectively toxic metal compounds (such as compounds of silver for infections resulting from burns and bismuth in fighting *Helicobacter pylori*) have long been used as antimicrobial compounds, antiseptics, and disinfectants (1). It is therefore paradoxical that metal compounds are the most abundant class of compounds for delivering carbon monoxide (CO) for *therapeutic* purposes in higher organisms. Although CO is a respiratory poison, it has “come of age” since the discovery that CO is a cytoprotective and homeostatic molecule and a vasodilator, anti-inflammatory, anti-apoptotic, and anti-proliferative agent (2–4). The biological chemistry of CO is relatively simple (when compared with O_2_ and the “gasotransmitters” NO and H_2_S) (5, 6). Its most important property is reaction with metals, famously ferrous heme proteins, although some heme-independent reactions are known, such as binding to iron in hydrogenases (7) and to binuclear copper sites, for example in hemocyanins (8). In CO dehydrogenase, which oxidizes CO to CO_2_, CO interacts with the nickel ion in one of the metalloclusters (“C-cluster”) (9). Here we review the effects of CO and CO-releasing molecules (CORMs)^2^ on microorganisms, experiments that demonstrate the potential of CORMs, and highlight problems and prospects.

## Development and Applications of CORMs

Resistance to antibiotics now threatens the effective prevention and treatment of microbial infections (10). This scenario is not an apocalyptic fantasy, and has promoted research into the development of new antimicrobial agents. CORMs, originally developed for therapeutic delivery (3, 4), have recently been investigated for their antimicrobial activities, initially presumed to be mediated by CO. If the delivery of CO to targets could be controlled and enhanced, it might be toxic to microorganisms; indeed, CO-supplemented gas atmospheres preserve meat from bacterial spoilage (11). However, microbes may also be relatively insensitive to the gas. Airborne bacteria survive high urban CO concentrations (12), and bacterial cultures may be bubbled with the gas (13); 250 ppm of CO is not toxic (14). Furthermore, CO *per se* is not selectively toxic to microbes; it is tolerated at about 3 mg/kg for 1 h in humans, and no toxic effects are evident in animal models at efficacious doses of the gas (when carbonmonoxyhemoglobin levels reach ∼20%) (4).

The key to the use of CORMs as antimicrobials is that they are far more toxic to microbes than is CO, but the basis of this toxicity is poorly understood. Mann (3) authoritatively reviews the discovery and development of CORMs. Early biological studies investigated binding to heme proteins, vasodilation, inhibition of NO production by macrophages (because CO deactivates inducible NO synthase while activating guanylyl cyclase), and survival of animals after organ transplantation (3, 15). Antimicrobial effects were not considered. Numerous CORMs have been reported and synthesized, but here and in Table 1, we describe only those that have been used against microbes or hold particular promise (16–26). Two ruthenium compounds have been extensively used: CORM-2 and CORM-3. The former has long been commercially available, but the latter has only recently been marketed. Although CORM-2 is soluble in dimethyl sulfoxide, the outstanding merit of CORM-3 is water solubility (27, 28). However, it has complex solution chemistry, and many aspects of its biological fate and CO release remain unresolved. In water, CO release is slow so that solutions can be prepared and administered with ease, but CORM-3 releases CO rapidly in the standard assay that uses ferrous myoglobin as acceptor, leading to the description of CORM-3 as a rapid CO releaser (27) (but see below).

**TABLE 1:**
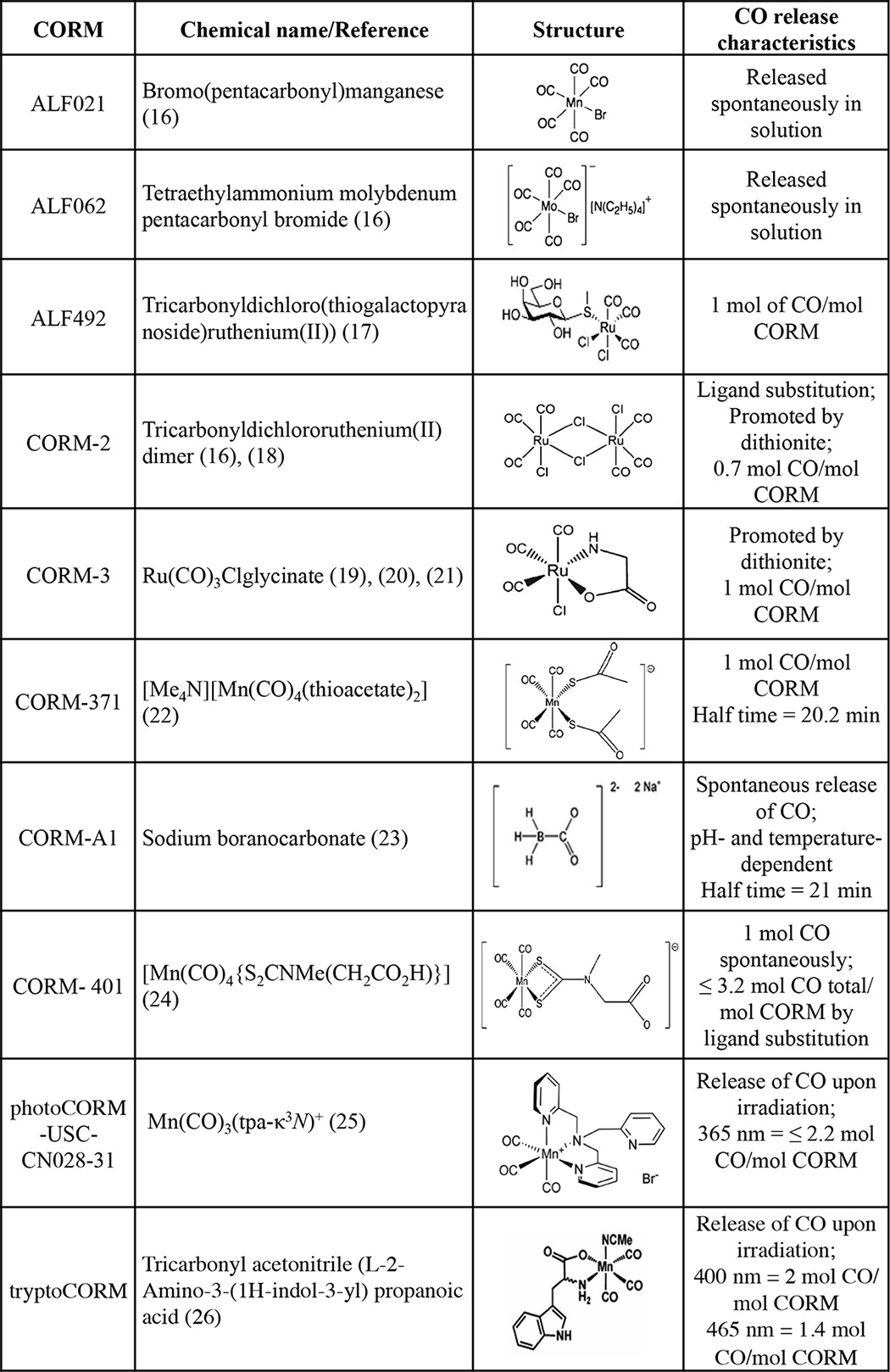
**CORMs referred to in this review**

Other CORMs are covered below where they have been used as antimicrobial agents. Newer compounds with desirable therapeutic effects are constantly appearing, but few have been tested microbiologically (29, 30). Of particular interest are CORMs in which the CO release can be precisely controlled both spatially and temporally, either by triggering the inactive “prodrug” with light (photoCORMs) (31) or by enzyme activation (32).

## Analytical Methods as a Bottleneck in Understanding CORM Toxicity

CO is generally assayed in environmental, clinical, or experimental situations by measuring the characteristic absorbance spectrum on reaction with myoglobin (above), or by GC-thermal conductivity detection (33, 34), solution IR spectroscopy (35), gas-phase IR absorption spectroscopy (36), attenuated total reflection IR spectroscopy of a metal carbonyl (37), chromogenic probes (38, 39), or metal oxide semiconductors (40). The CO electrode produced by World Precision Instruments is potentially useful but has been little used to date (22). An amperometric microsensor simultaneously measures NO and CO in mouse kidneys (40), but such electrodes are currently unsuitable for detecting and quantifying CO released inside microbes by CORMs.

The standard laboratory method for detecting CORM-derived CO *in vitro* is the myoglobin assay (18) in which the liberated CO reacts with ferrous myoglobin to give a distinct CO adduct. The method compares favorably with GC-thermal conductivity detection of CO (41). Refinements to the myoglobin assay were proposed (42), but we demonstrated that it is the reducing agent for myoglobin, sodium dithionite, that promotes CO release (43); CO is not released from CORM-3 in the absence of the reductant (43). It might be explained by the fact that dithionite is not pure and contains a significant quantity of sulfite, which is in equilibrium with sulfur dioxide, a good ligand for transition metals. This fits with the observation that, on dissolution in buffers in a closed vial, only CO_2_, resulting from the water-gas shift reaction, can be detected (by GC) (33). The mechanism of CO release from CORM-3 remains unknown as its chemistry is complex (28), but decomposition products of CORM-3 react with exposed His residues on protein to give metalloproteins that spontaneously release CO (44). Thus, in biological situations where dithionite (or sulfite, metabisulfite, or perhaps other species) are absent, the myoglobin assay overestimates the rate of CO release. Likewise, CORM-3 does not release CO to the purified flavohemoglobin (Hmp) when reduced with NADH but does so in the presence of dithionite (45). These findings probably explain the discrepancy noted between the myoglobin assay and the CO electrode (22), previously attributed to the need for certain CORMs to interact “with biological components to trigger the release of CO” (22). An alternative assay that obviates the need for dithionite uses oxyhemoglobin (43). Such globin assays could in principle be applied to CO assays within bacteria; indeed *Escherichia coli* Hmp expressed at high copy number is a sensitive monitor of CO liberated inside bacteria from CORMs (45).

Newer methods with unrealized potential include FTIR and photothermally induced resonance to detect an organometallic carbonyl compound (not a CORM) in breast cancer cells (46). More promising is Raman microspectroscopy to detect a manganese CORM [(Mn(tpm)(CO)_3_]Cl (tpm = tris(1-pyrazolyl)methane) in colon cancer cells (47). A genetically constructed fluorescent probe (COSer) comprises the CO binding selectivity of CooA, a dimeric CO-sensing heme protein from *Rhodospirillum rubrum*, and a fluorescent peptide to report conformational changes on binding CO (48). Transfection of HeLa cells with COSer allowed intracellular imaging of CO after treatment with CO or 1–10 μm CORM-2. A new fluorescent probe (COP-1) based on palladium-mediated carbonylation allowed selective CO detection in cells after CORM-3 treatment (49). COP-1 has also been used *in vitro* to demonstrate CO release from a photoCORM in the presence of endothelial cells (35). Zobi *et al.* (50) have shown via synchrotron FTIR spectromicroscopy that a photoactivated CORM conjugated to vitamin B_12_ is taken up by fibroblasts. A photoCORM that is also luminescent could be tracked by confocal fluorescence microscopy (51). These methods have not been tested in bacteria, but the attainable spatial resolution appears at present inadequate for subcellular localization.

## CO Metabolism in Microorganisms: Implications for Pathogenesis

To understand the possible mechanisms of action of CORMs, it is clearly important to appreciate how CO *per se* impacts on microorganisms. DNA replication is inhibited by CO (52), and the inhibition by CO of respiratory oxidases and globins at heme targets has been known since the days of Warburg and Keilin (reviewed in Ref. 53). However, CO also binds to the di-iron site in bacterial NO reductases (54, 55) and to iron, copper, and nickel sites in certain microbial proteins, notably CO dehydrogenase (see above).

The relationship between CO and disease is complex, but clues come from the observation that cigarette smoking and CO, a component of smoke, have anti-inflammatory effects against ulcerative colitis (56). However, the major CO source in mammals is CO endogenously produced by heme oxygenase (HO)-1 (57). Several bacteria also possess HO enzymes that function to degrade heme that is imported for use as an iron source (58, 59). HO activity contributes to pathogenesis in certain bacteria by scavenging iron from heme (58, 60).

There is extensive literature on sensing of gases (O_2_, NO, CO) by mycobacteria and its role in dormancy. *Mycobacterium tuberculosis* infection of macrophages and mice induces host HO-1 expression (61). The CO thus produced, together with iNOS-derived NO, stimulates expression (via the heme two-component sensor kinases DosS and DosT and the cognate response regulator DosR) of the bacterial dormancy regulon, a group of about 50 genes with diverse functions (61, 62). A recently described CO resistance gene (*cor*) in *M. tuberculosis* appears important in dictating the outcome of the host-bacterium battle; the virulence of a *cor* mutant is attenuated in a mouse model of tuberculosis. Expression of the Cor protein in *E. coli* is claimed to rescue it from CO toxicity, but the resistance demonstrated was to CORM-2 not CO (63).

The HO (Hmx1) of the pathogenic yeast *Candida albicans* and its product, CO, also contribute to pathogenesis (64); mutagenesis of the *HMX1* gene results in decreased virulence in murine candidiasis, whereas exposure of mice to therapeutic levels of CO increases *C. albicans* virulence. Inhaled CO partially reverses the virulence defect of the null strain, and so the data are consistent with CO-mediated suppression of acute host inflammatory responses (64).

## Heme Oxygenases of Mammalian Cells: Implications for Infection

Mice deficient in HO-1 are susceptible to oxidant-induced tissue injury, but administration of CO to animals exposed to endotoxin decreases inflammation. HO-1- or CORM-2-derived CO rescues mice from lethal endotoxemia and sepsis (65). However, the role of CO in tackling a pathogen is less clear (66, 67). Indeed, suppression of inflammation might compromise the immune system. Otterbein *et al.* (68) showed that CO gas enhances phagocytosis, and Chung *et al.* (69) showed that CO derived from HO-1 enhanced the host defense response to polymicrobial sepsis in mice and contributed to bacterial clearing by stimulating phagocytosis.

Enterohemorrhagic *E. coli* (EHEC) stimulate the rapid inducible expression of the human enterocyte *HMOX-1* gene that encodes HO-1, and its activity is a critical modulator of the innate immune response (70). Because HO-1 activity inhibits iNOS induction, EHEC effectively suppresses NO generation, and thus host antimicrobial activity. The CO donor CORM-2 also inhibited iNOS mRNA expression, thus identifying CO, not bilirubin (another product of HO-1 activity), as the effective species (but see caveats below regarding the non-equivalence of CORMs and CO). Up-regulation of HO-1 was shown to offer protection in mice against infection by *Mycobacterium avium* or *M. tuberculosis*, whereas HO-deficient mice were more susceptible (71). Thus, HO-1 may be an important cytoprotective protein in sepsis and inflammation.

CO is also implicated in the pathogenesis of *Clostridium difficile*. Inhibition of host HO activity by administering Zn protoporphyrin IX to mice exacerbated the histopathological alterations elicited by *C. difficile* toxin A; conversely, pretreatment of mice with a CO donor (dimanganese decacarbonyl) reduced the effect (60).

In a recent study, enteric microbiota isolated from pathogen-free mice induced production of HO-1 in colons of wild-type mice but not in colitis-prone interleukin (*Il)10*^−/−^ animals (72). However, pharmacological induction of HO-1 by Co(III) protoporphyrin IX chloride protects interleukin^−^ mice from microbiota (*Salmonella enterica* serovar Typhimurium)-induced colitis. Moreover, HO-derived CO reduced the numbers of live bacteria recovered from various organs, whereas knockdown of HO-1 in macrophages impaired bactericidal activity. Thus, HO-1 and CO ameliorate intestinal inflammation through promotion of bacterial clearance, in part explained by promoting bactericidal activities of macrophages (72, 73).

Recently, Wegiel *et al.* (14) have proposed that ATP, acting as a pathogen-associated molecular pattern, which is recognized by innate immune cells, is released from viable bacteria in the presence of CO and triggers activation of the macrophage, inflammasome. and IL-1β secretion. Curiously, it is suggested that an oxidase binds CO “to compel ATP generation much like that observed in the ATP synthase mutant” (14). However, Gram-negative bacteria are not known to possess periplasmic ATP or to have mechanisms for secretion, so the observed effect is poorly understood.

## The Antimicrobial Effects of CO and CORMs *in Vitro* and *in Vivo*

**FIGURE 1.**
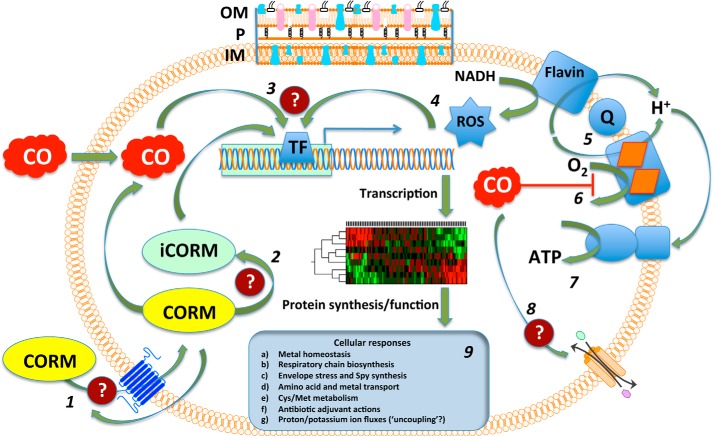
**Sites of action and cellular consequences of bacterial exposure to CO and CORMs.** Outcomes are generalized and pooled from the reported effects of various metal carbonyl compounds (for details, see the text). The bacterial inner membrane (IM) is shown together with the outer membrane (OM) and periplasm (*P*) at the top only. The OM is considered freely permeable to CORMs; transport events are therefore shown through the IM only. **1**, CORMs enter bacteria by unknown pathways and driving forces; CO enters by diffusion down concentration gradients. CORMs may in principle be exported. **2**, CORM releases CO intracellularly, leaving a metal-coligand fragment or iCORM. **3**, transcription factors (TFs) sense CO, CORM, and iCORM, leading to global transcriptional effects and modified protein profiles. **4**, TFs are also activated by ROS that may be generated directly by cellular CORM chemistry or from leakage of reducing equivalents from respiratory chains. **5**, a typical simplified bacterial aerobic respiratory chain is shown comprising a flavin-containing NADH dehydrogenase, a ubiquinone (*Q*) pool, and a terminal heme-containing quinol oxidase. **6**, CO binds to the oxidase active site, competing with oxygen and blocking respiration. **7**, ATP generation via ATP synthase is compromised. **8**, CO (or CORM, not shown) may directly or indirectly interact with IM transporters. **9**, diverse cellular responses to CO and CORM exposure are reported. Four outstanding areas of uncertainty are highlighted (*question marks*): transport of CORMs into (or out of) cells; intracellular mechanisms of CO liberation from CORMs; modification of TF function and gene expression by CORMs; and effects of CO and CORMs on membrane transporters.

In many respects, CO is an attractive candidate for an antimicrobial molecule; it is rarely metabolized and “stable,” is adequately water-soluble, traverses cell membranes (5), and is a molecule that is naturally generated in mammals, plants, and certain microorganisms by HO (supplemental Table 1). There is a rapidly growing literature on the diverse antimicrobial effects of CORMs on bacteria (Fig. 1). Nobre *et al.* (16) first described the use of CORMs as antimicrobial agents. CORM-2 and CORM-3 and compounds from Alfama, Inc. (ALF021, bromo(pentacarbonyl)manganese, and ALF062, tetraethylammonium molybdenum pentacarbonyl bromide) (Table 1) were tested against laboratory strains of *E. coli* and *Staphylococcus aureus* (16). For example, killing of greater than 20% was achieved within 1 h with 250 μm CORM-2, and more variable killing was achieved with 400 μm CORM-3. Control experiments with hemoglobin to sequester CO and the use of inactive forms of the CORMs or solvent-only controls suggested that CO release was the major cause of killing, yet a flux of CO gas (∼1 mm dissolved concentration) was markedly less effective than the CORMs. Interestingly, CO was not detected in media to which the CORMs were added, implying that CO release occurs only intracellularly or that the CO liberated extracellularly escapes from the culture.

Three important studies indicate the potential for CORM-elicited antimicrobial effects in animal models. Chung *et al.* (69) showed that CO from HO-1 enhanced the response to sepsis in mice and stimulated phagocytosis, an effect mimicked by injection of CORM-2. Second, CORM-2 and CORM-3 were effective in protecting immunocompetent and immunocompromised mice when injected following *Pseudomonas aeruginosa*-induced bacteremia (20), but CORM-371 was not (22). The data suggest a direct bactericidal action rather than stimulation of phagocytosis. Third, activity of ALF492 (tricarbonyldichloro(thiogalactopyranoside)Ru(II)) (Table 1) was demonstrated (17) in mice against the protozoan parasite *Plasmodium falciparum*; the injected compound protected mice against experimental cerebral malaria and acute lung injury without formation of carbonmonoxyhemoglobin. The protective effect was CO-dependent, and the CORM elicited expression of HO-1, thus amplifying the protection. ALF492 was also shown to be an adjuvant to the established antimalarial compound artesunate (17).

However, most recent studies have used *in vitro* methods and cast doubt on our understanding of the fundamental modes of action, especially the suggestion that CORMs exert antimicrobial activities solely through CO release. Several authors have reported that CORMs are more effective antimicrobial agents than is CO (16, 21). For example, 100 μm CORM-3 was effective against *P. aeruginosa in vitro* (20), but CO gas (∼860 μm) was not. Importantly, even 10 μm CORM-3 was effective against antibiotic-resistant clinical isolates but was not inhibitory to macrophage survival.

Recently, CORMs that release CO only on illumination have been developed and tested as antimicrobial agents. The first such study describes a manganese CORM (Table 1) that acts as a stable prodrug in the dark, whereas 365 nm illumination leads to CO release to myoglobin (25). Only after irradiation is the compound toxic to *E. coli*, in which CO-ligated terminal oxidases can be detected following internalization of the compound. This compound has the advantage of a well defined inactivated form of CORM (iCORM) (25). Similarly, a tryptophan-derived manganese-containing complex (tryptoCORM) that releases 1.4 mol of CO on irradiation at 465 nm, and 2 mol at 400 nm, is toxic to *E. coli* but not to macrophages (26).

Concerns over the inexorable spread of antibiotic resistance and the paucity of new antimicrobial drugs have led to studies not only of CORMs as antimicrobials in their own right against antibiotic-resistant clinical isolates (20, 74), but also as adjuvants to established antibiotics, a common practice in clinical therapy (*i.e.* combination therapy) (supplemental Fig. 1). In one study, sub-lethal doses of CORM-2 were combined with metronidazole, amoxicillin, and clarithromycin and found to potentiate antibiotic effects on clinical isolates of *H. pylori* (75). Two mechanisms of action were reported: inhibition of respiration and of urease activity. CORM-2 decreased the measured minimal inhibitory and minimal bactericidal concentrations for all antibiotics. Similarly, CORM-2 acts as an adjuvant to tobramycin against *P. aeruginosa* biofilms (76). In neither of these studies was it reported whether the effects of CORM-2 and antibiotics together were truly synergistic or merely additive, as assessed by standard fractional inhibitory concentrations (77). However, these potentiating effects observed with CORMs have not been reported to our knowledge with CO gas, although NO and H_2_S have been shown to confer some defense against antibiotics (78).

## Transcriptomic and Global Impacts of CORMs

Transcriptomic approaches have been highly informative and emphasized the complexity of the CORM response. In the first study (21), batch cultures of *E. coli* were used to explore exposure to sub-inhibitory (30–100 μm) concentrations of CORM-3, aerobically and anaerobically. The down-regulation of operons encoding key respiratory complexes (cytochrome *bo*′ and several dehydrogenases) was striking. Interestingly, the *cydAB* genes encoding cytochrome *bd*-I, an inhibitor-resistant terminal oxidase with a high oxygen affinity, were slightly up-regulated. The genes most highly up-regulated were involved in metal homeostasis, especially *spy*, which encoded a periplasmic stress-response chaperone. Probabilistic modeling of the comprehensive datasets (21) identified global transcription factors that are potential CO targets or sensors, notably the respiratory metabolism regulators ArcA and Fnr. However, a similar study using 250 μm CORM-2 (partly bactericidal within 30 min (16)) revealed (79) a gene set with few similarities to the CORM-3 study, but up-regulation of *spy* and down-regulation of some respiratory operons were observed.

A more rigorous and reproducible approach to transcriptomics is provided by chemostat (continuous) culture in which all growth conditions, including growth rate, are maintained over long periods, thus avoiding growth rate-dependent changes in gene expression (80). Mclean *et al.* (81) used not only CORM-3 but also the inactivated iCORM-3 (from which negligible CO release can be shown) to dissect the effects of CO release and other consequences of the *E. coli* response in a chemostat. Transcriptomics revealed that the response to iCORM-3 is lower than to CORM-3, but that numerous processes are affected by both compounds, including energy metabolism, membrane transport, motility, and the metabolism of sulfur-containing species, including cysteine and methionine.

There is controversy regarding the roles of reactive oxygen species (ROS) and antioxidants in the antibacterial effectiveness of CORMs; the evidence in favor is given in Ref. 59. It is established that inhibition of bacterial oxidase activity by CO can lead to higher ROS levels (82), for example from exposed flavins in NADH dehydrogenase (83). However, Tavares *et al.* (84) propose the direct involvement of ROS in the toxicity of CORM-2 and ALF062 to *E. coli*; both promote the production of reactive oxygen species, an effect blocked by antioxidants. Mutations in superoxide dismutase or catalase exacerbated CORM toxicity, and CORM-2 induced expression of the DNA repair/SOS system *recA* and raised levels of free iron in cells. In contrast, treatment of *P. aeruginosa* with three CORMs did not change ROS production (22).

Certain antioxidants (*N*-acetylcysteine (NAC) and ascorbic acid) suppress H_2_O_2_ levels, and NAC, cysteine, and reduced (but not oxidized) glutathione reverse CORM-3-mediated inhibition of bacterial growth and respiration (20, 81). Glutathione and cysteine also prevented killing of *H. pylori* by CORM-2, but ROS could not be detected and ascorbic acid did not prevent the antimicrobial effect of CORM-2 (75). Thus, the basis of the effects of these sulfhydryl compounds remains poorly understood but is important because many are intracellular compounds and might promote or modulate CO release *in vivo* (81). Significantly, the effects of antioxidants on CORM toxicity may be linked, not only to counteracting the intracellular toxic effects, but also to the uptake of the CORM. Jesse *et al.* (85) found that NAC, widely used to abrogate CORM effects, not only protected respiration from CORM-2 or CORM-3 but also dramatically reduced (5–8-fold) CORM uptake.

The transcriptomic evidence is contradictory. Many genes implicated with intracellular redox stress were reported in *E. coli* by some (79) but not all (21) authors. The genes *spy*, *spb*, *metF*, and *htpX* seen by us (21) are described in Ref. 59 as “associated with the generation of intracellular oxidative stress.” However, the up-regulation of *spy* (the most dramatically changed gene: 26–100-fold (21), not 3-fold as reported in Ref. 59)) is attributed not exclusively to oxidative stress but to hypochlorite-induced membrane disruption (86).

## How Significant Is Respiratory Blockade in Determining CORM Effectiveness?

Cellular respiration is inhibited by CO gas *in vitro* and in cells via endogenous HO activity (87, 88). Although reaction of CORM-derived CO with intracellular ferrous hemes has been reported consistently (*e.g.* Refs. 21, 45, and 82), and functionally distinct oxidases have differential sensitivities to CORMs (85), inhibition of respiration is not the only factor affecting the bactericidal activity of CORMs (22). CORMs may be toxic under anoxic conditions in the absence of respiration (16, 20, 21). Indeed, in mitochondria, CORMs may inhibit respiration (87, 89) or not (90–92). The reported uncoupling of mitochondrial respiration by CORM-3 (deduced from stimulated oxygen consumption rates) (90–92) and by CORM-401 in cardiomyocytes (93) is relevant to bacteria because CORM-3 at low doses also stimulates respiration in *E. coli* (94). However, classical uncoupling appears not to be the cause because proton translocation quotients and proton backflow rates are unaffected by CORM-3 (94). The stimulatory effects may arise from reaction of CO or CORMs with membrane channels as described in mammalian cells (95–97).

## What Is the Mechanism of CORM Activity against Microorganisms?

Wherever an answer to this key question has been sought, investigators have found that bacteria accumulate CORMs (16, 21, 81, 85), that CO is bound to identifiable targets (*i.e.* heme proteins), and CO causes global changes in gene expression and cell function (Fig. 1). Furthermore, CO gas (as evidenced from data with HO-derived CO *in vivo*; see above) also perturbs microbial behavior. However, although CORMs were originally developed for safe and reproducible delivery of CO in mammals, the evidence to hand, summarized above, makes it improbable that CO delivery alone is the sole basis of the antimicrobial effects of CORMs. What evidence supports this bold claim? (*a*) Saturating solutions of CO gas barely perturb bacterial growth. (*b*) Bacteria demonstrate multiple transcriptomic changes to CORM-3 that cannot be understood in terms of known CO biochemistry. (*c*) Bacteria respond to iCORM-3 from which no, or negligible, CO release can be demonstrated *in vitro*. (*d*) Critically, cells lacking all hemes are also inhibited by CORM-3 and reveal multiple transcriptomic changes (101). (*e*) Finally, other compounds of Ru are taken up and have antimicrobial properties, although they are not CORMs (*e.g.* Refs. 1 and 98). We have suggested (94) that a CORM functions as a “Trojan Horse,” in which the metal carbonyl is the “horse,” delivering a cargo of toxic CO; it is equally conceivable that the toxic cargo is the metal fragment and that CO potentiates uptake.

## Future Prospects

Realizing the future potential for CORMs relies on greater understanding of the modes of action of current CORMs and the development of improved compounds with clinical compatibility, for example by making biocompatible CO carriers (99). In the post-antibiotic era, there appears to be potential for adjuvant/combination therapy in which CORMs can minimize usage of established antibiotics or reduce the concentrations needed to treat antibiotic-resistant “superbugs.” Apart from methodological advances in detecting CO, a “CO-quenching” agent would allow the essential dissection of the antibacterial roles of the CO *per se* and the CORM; a water-soluble complex has been tested as a CO “stripper” in a rat model (100). Other areas of focus should be improved iCORMs that can be reproducibly prepared and whose chemistry is understood, a study of the potential for microbes developing resistance to CO or CORMs, and a better understanding of the biological chemistry of non-heme CO targets.

## Supplementary Material

Supplemental Data

## Footnotes

2 The abbreviations used are:

CORM: CO-releasing molecule
iCORM: inactive form of CORM from which CO release cannot be detected
HO-1: heme oxygenase 1
NAC: *N*-acetylcysteine
ROS: reactive oxygen species
iNOS: inducible NOS
photoCORM: photoactivatable CORM
tryptoCORM: tryptophan-derived manganese-containing complex
IM: inner membrane
OM: outer membrane
TF: transcription factors.
