# Supplementary material for: CO-releasing Metal Carbonyl Compounds as Antimicrobial Agents in the Post-antibiotic Era
Source: J Biol Chem. 2015 Jun 8;290(31):18999–9007. doi: 10.1074/jbc.R115.642926 (PMC4521022; doi:10.1074/jbc.R115.642926)
Supplement: Supplemental Data [file supp_290_31_18999__index.html]

CO-Releasing Metal Carbonyl Compounds as Antimicrobial Agents in the Post-Antibiotic Era — CO-releasing Metal Carbonyl Compounds as Antimicrobial Agents in the Post-antibiotic Era — MINIREVIEW: CORMs as Antimicrobial Agents — Supplemental Data 

# CO-releasing Metal Carbonyl Compounds as Antimicrobial Agents in the Post-antibiotic Era

## Supplemental Data

- Supplemental material for teaching purposes (.pdf, 1.0 MB) - Supplementary Table and Figure for teaching purposes only
