# Supplementary material for: CO-releasing Metal Carbonyl Compounds as Antimicrobial Agents in the Post-antibiotic Era
Source: J Biol Chem. 2015 Jun 8;290(31):18999–9007. doi: 10.1074/jbc.R115.642926 (PMC4521022; doi:10.1074/jbc.R115.642926)

SUPPLEMENTARY TABLE 1

CO and CORMs as antimicrobial agents: a comparison

	**Pros**	**Cons**
**CO gas**	Stability	Ultimately toxic to almost all organisms
	Adequate water solubility	Low toxicity against bacteria
	Facile diffusion across membranes	Difficult handling and administration
	Produced intracellularly by heme oxygenases	
**CORMs (generalized properties)**	Toxicity against bacteria (e.g. CORM-2 and CORM-3)	Complex chemistry
	Easy handling and administration	Lack of knowledge on biological fates
	CO release can be controlled (e.g. PhotoCORMs and enzyme-activated CORMs)	Only some CORMs have been tested microbiologically
	New CORMs with desirable therapeutic effects are appearing	

SUPPLEMENTARY FIGURE 1

Antibiotics and CORMs have distinct cellular targets

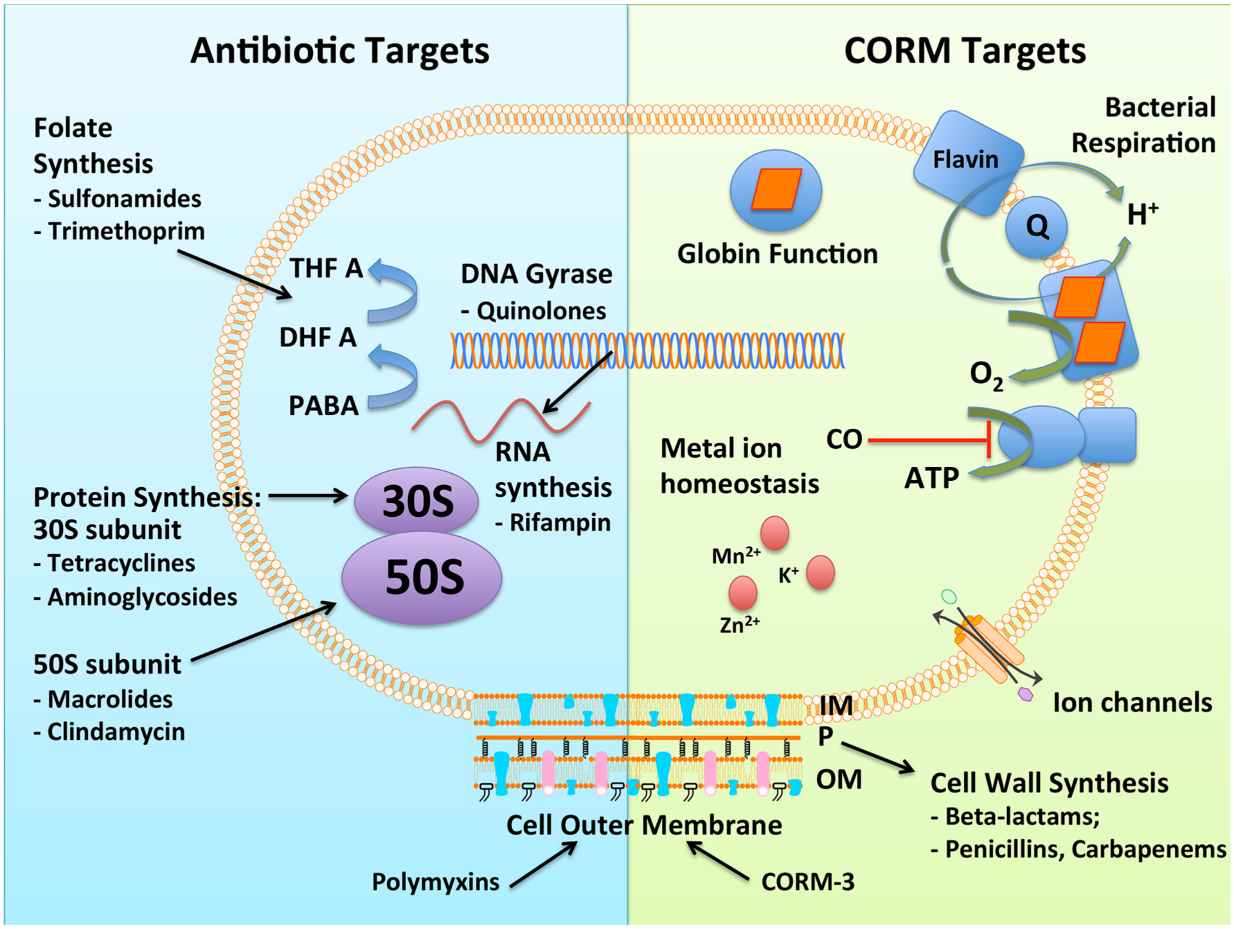

Supplement: Supplemental Data [file supp_R115.642926_jbc.R115.642926-1.pdf]
